# Correction: Management of relapsed and refractory multiple myeloma: novel agents, antibodies, immunotherapies and beyond

**DOI:** 10.1038/s41375-019-0410-3

**Published:** 2019-03-06

**Authors:** C. S. Chim, S. K. Kumar, R. Z. Orlowski, G. Cook, P. G. Richardson, M. A. Gertz, S. Giralt, M. V. Mateos, X. Leleu, K. C. Anderson

**Affiliations:** 1 Department of Medicine, Queen Mary Hospital, The University of Hong Kong, Hong Kong, Hong Kong; 2 Department of Medicine, Mayo Clinic at Rochester, Rochester, MN USA; 3 Department of Lymphoma/Myeloma, Division of Cancer Medicine, The University of Texas MD Anderson Cancer Center, Houston, TX USA; 4 Haematology & Myeloma Studies, Section of Experimental Haematology, Leeds Institute of Cancer and Pathology, University of Leeds, Leeds, UK; 5 Department of Medical Oncology, Dana-Farber Cancer Institute, Harvard Medical School, Boston, MA USA; 6 Department of Medicine, Memorial Sloan Kettering Cancer Center, New York, USA; 7 Department of Haematology, University Hospital of Salamanca, Salamanca, Spain; 8 Hopital La Mileterie, part of the Academic Hospital of Poitiers (CHU), Poitiers, France

**Correction to:** **Leukemia** 32, 252–262 (2018);

10.1038/leu.2017.329;

published online February 2018

Following the publication of this article the authors noted that the MRD data under the Table 1 column “Remark” of Aspire should go to that of Pollux. The authors wish to apologize for any inconvenience caused. The corrected table is attached to this correction.

Table. Major clinical trials of next generation PIs (carfilzomib, Ixazomib), next generation IMiDs (pomalidomide) and monoclonal antibody.

